# The Impact of Bacterial Leaf Blight Disease (*Pantoea agglomerans*) on Grain Yield and Nutritional Quality of Oat

**DOI:** 10.3390/microorganisms13010141

**Published:** 2025-01-11

**Authors:** Ruochen Zhang, Jianjun Wang, Longhai Xue, Malik Kamran, Yue Wang, Xuekai Wei, Guiqin Zhao, Chunjie Li

**Affiliations:** 1 State Key Laboratory of Herbage Improvement and Grassland Agro-Ecosystems, Key Laboratory of Grassland Livestock Industry Innovation, Ministry of Agriculture and Rural Affairs, Engineering Research Center of Grassland Industry, Ministry of Education, Gansu Tech Innovation Center of Western China Grassland Industry, College of Pastoral Agriculture Science and Technology, Lanzhou 730020, China; zhangrch2021@lzu.edu.cn (R.Z.); jianjun@lzu.edu.cn (J.W.); xuelonghai@lzu.edu.cn (L.X.); malik@lzu.edu.cn (M.K.); 220220901970@lzu.edu.cn (Y.W.); weixk@lzu.edu.cn (X.W.); 2 Pratacultural College, Gansu Agricultural University, Lanzhou 730070, China; zhaogq@gsau.edu.cn

**Keywords:** *Pantoea agglomerans*, leaf blight disease, grain yield, nutritional quality, oat

## Abstract

As an important cereal and feed crop, oat has significant economic value and is widely cultivated throughout the world. However, leaf diseases have become a crucial factor limiting the increase in oat grain yield and the optimization of its nutritional quality. Among these, the bacterial leaf blight disease (LBD) caused by *Pantoea agglomerans* has been an emerging and prevalent oat disease in Northwest China in recent years and has become a major challenge for oat cultivation in this region. This study was designed to investigate the effects of LBD on grain yield and nutritional quality of two common oat varieties, i.e., *Avena nuda* “Baiyan 2” (B2) and *A. sativa* “Baiyan 7” (B7), in greenhouses. The results showed that after infection causing LBD, the growth, grain yield and nutritional indexes (except the fiber content) of B2 and B7 were significantly reduced (*p* < 0.05), with grains per spike, thousand grain weight, protein, and β-glucan reduced by 14.2%, 5.5%, 12.9% and 21.5%, respectively. In contrast, the average fiber content of the infected oats increased by 8.4%. In addition, both with and without infection, the grain yield of B7 was higher than that of B2, while the nutritional quality of B2 seeds was superior to that of B7 seeds. This study provides a scientific basis for LBD control and the variety selection of oat, promoting the sustainable development of the oat industry.

## 1. Introduction

Oat is a most cropped cereal and economically important fodder crop cultivated throughout the world, especially in arid and semiarid regions, such as Northern Europe, North America, Australia, Canada and China [1,2]. Meanwhile, it is also a major spring crop, following wheat, rice, maize, barley and sorghum, and its grains are usually consumed as a healthy food and livestock feed because of its high levels in protein, β-glucan, minerals, antioxidants, dietary fiber, etc. [3,4,5]. Some studies reported that oat contains a minor protein, “prolamine”, and other components such as tocols, alk(en)ylresorcinols, avenanthramides, phenolic acids and their derivatives that have antioxidant properties and may contribute to human health [4,6,7]. Thus, with the increasing human needs for healthy food and livestock need for good fodder, oat has received substantial attention for its excellent edible and feeding quality as well as major economic benefits and values for human health and livestock nutrition [1]. The improvement of oat yield and nutritional quality has great economic and social importance for human consumption and livestock nutrition.

Like other cereals, oat growth and production are also subjected to a number of fungal, bacterial and viral diseases that may damage or destroy oat growth and fields [8]. Diseases such as stem rust (*Puccinia graminis*) [9], bacterial leaf blight (*Pseudomonas* spp.) [10,11], smut (*Ustilago* spp.) [12], crown rust (*Puccinia coronata*) [13], leaf blotch (*Pyrenophora avenae* or *Bipolaris* spp.). [14,15], Fusarium head blight (*Fusarium* spp.) [16], barley yellow dwarf virus (BYDV) [17] and powdery mildew (*Blumeria graminis*) [18] are the main diseases affecting oat production in most places. The occurrence and development of these diseases tend to limit oat performance and cause severe yield losses and are also responsible for a progressive decline in worldwide oat production.

Our previous survey indicated that bacterial leaf blight disease (LBD) caused by *Pantoea agglomerans* is a recently emerged and prevalent disease of oat (*A. sativa*) in Huan county Qingyang City, Gansu Province, in northwestern China [19]. *P. agglomerans* is a Gram-negative bacterium, widely distributed in agricultural and natural environments, affecting plants, flowers, seeds, fruits, water, soil, humans and animals [20,21]. This bacterium is a pathogen of plants and humans and causes an opportunistic infection [22]. In most cases, this species can incite soft tissues bone and joint infections after penetration of the skin of human beings, which can develop into bacteremia or neonatal sepsis [21,22,23]. Furthermore, *P. agglomerans* can also cause leaf spots, leaf blights and rot diseases in many plants as a plant pathogen, such as boll rot in cotton [24], leaf spot in the Araceae family [25], top or stem rot in maize [26] and LBD in onions [27], rice [28], walnuts [29], pepino melon [30], maize and sorghum [31]. It was shown that *P. agglomerans* caused a decrease in crop yield and nitrogen-free extract content and a decrease in the nutritive value and feed intake of silage [32]. However, it has also been reported that *P. agglomerans* has a positive influence on the growth of wheat and rice crops [33]. Currently, it is unclear if these diseases caused by *P. agglomerans* have direct influences on the growth, grain yield and nutritional quality of oat; particularly, the effects of LBD on oat grain production are not known. Additionally, it is not known if the severity of LBD varies based on factors such as infection degree of *P. agglomerans* and disease index, which further complicates the control of LBD.

Understanding the effects of LBD on oat and their potential mechanisms is crucial for reducing the loss of grain yield and nutritional quality of oat and is essential to ensure a sustainable oat production in the face of LBD challenges. The current study focuses on the dual impacts of LBD on the feeding and nutritional value of oats; it was conducted to further understand and explore the impacts of LBD (*P. agglomerans*) on grain yield and nutritional quality of covered and naked oat. The study was aimed to evaluate the hypotheses that (1) LBD negatively affects the grain yield and nutritional quality of oat; (2) these negative effects of LBD on covered and naked oat plants are different; (3) the effects increase with the development of LBD.

## 2. Materials and Methods

### 2.1. Plant Material

The two common oat varieties “Baiyan 2” (B2) and “Baiyan 7” (B7), widely planted in northern China, were used in this study. The seeds of *A. sativa* (B7) and *A. nuda* (B2) were harvested from Baicheng City, Jilin Province of China (45°37′ N, 122°48′ E, altitude 155 m), in 2018. The well-filled and healthy-looking seeds were collected and stored at a constant temperature of 4 °C at the Lanzhou Official Herbage and Turfgrass Seed Testing Centre, Ministry of Agriculture, Lanzhou, China, for further investigation.

### 2.2. Preparation of a Bacterial Suspension

The isolation and detection of the pathogenic bacterium causing LBD in oat were performed in our previous study [19]. A pure strain of *P. agglomerans* grown on nutrient agar (NA) for 24 to 48 h at 25 °C was suspended in sterile distilled water to obtain an optical density of approximately 108 (CFU)/mL measured using a Biolog turbidimeter. The prepared bacterial suspension was preserved at 4 °C for further analysis.

### 2.3. Seed Treatment and Seedling Cultivation

On 18 October 2019, B2 and B7 seeds were separately surface-sterilized with a 1% NaOCl solution for 10 min followed by 75% ethanol for 5 min, then thoroughly washed with sterile water 5 times and dried on sterile filter paper. The sterilized seeds were separately planted in 48-hole plastic seedling trays containing the same mass of sterilized vermiculite. The seedlings that grew two leaves were transplanted into plastic pots (diameter, 20 cm, height, 30 cm) filled with the same amount of sterilized medium (forest brown soil and natural soil in a w/w ratio of 1:2). The natural soil was collected from an experimental field of Lanzhou University. Each pot had only one seedling and equal initial water content. All the pots were randomly placed in a constant-temperature greenhouse (temperature, 22 ± 2 °C, humidity, 42 ± 2%) at the College of Pastoral Agriculture Science and Technology, Lanzhou University (annual mean maximum temperature of 27 °C in summer and minimum temperature of −7 °C in winter), The entire experimental process was conducted under natural lighting conditions, and the plants were watered as needed.

### 2.4. Bacterial Inoculation

A bacterial suspension was inoculated on the leaves of three-week-old healthy oat seedlings using the injection inoculation method (suspension infiltrated into the leaf tissues with a needleless syringe) in the greenhouse [34]. Seedlings inoculated with sterile distilled water were regarded as controls (CK), and a total of 180 pots were used for inoculating the suspension and water. All inoculated seedlings were placed in transparent polyethylene bags moistened with sterile distilled water at the beginning of the symptoms. Then, they all were randomly assigned to a location in the greenhouse and observed daily for three weeks for the development of disease symptoms. The diseased leaf samples were cut into small pieces (0.5 × 0.5 cm) from the lesion margins. The leaf pieces were surface-sterilized in 70% ethanol for 60 s, followed by treatment with a 1% NaOCl solution for 3 min. The samples were then rinsed twice with sterile water and air-dried. The dried samples were then macerated in 1 mL of sterile water for 20 min at 25 °C, and the bacterial suspension was streaked on NA and incubated at 25 °C for 24 to 48 h [19]. The re-isolation of bacteria from the inoculated plant lesions was carried out, and the bacteria were identified as *P. agglomerans* by colony morphology on NA and physiological and biochemical tests.

### 2.5. Disease Investigation

The average disease infection rate (ADR) and average disease index (ADI) of the inoculated seedlings were separately recorded 5 times in the jointing, flowering, and milking stages. The ADR and ADI were calculated by the following formula [35]: ADI = (sum [class frequency × score of rating class])/([total number of leaves investigated] × 4) × 100. ADR = number of leaves infected/total number of leaves investigated × 100. The disease rating criteria of LBD (*P. agglomerans*) were based on the system of bacterial LBD in rice (Table 1) [36,37]. So, this experiment involved 6 disease ratings × 2 oat varieties × 15 replicates per disease rating, resulting in a total of 180 pots used.

### 2.6. Grain Yield and Nutritional Quality Analysis

The parameters of panicle length, grains per spike, node number and reproductive branch number were measured in the full ripe stage, and mature seeds were also harvested from all inoculated plants. Then, moisture content, bulk density, thousand grain weight, ash, protein, fiber, ether extract, total starch, β-glucan, phytic acid, total phosphorus and Ca of different treatments were measured. The moisture content was determined using an oven at 105 ± 3 °C for 24 h, and the results were calculated and expressed as a percentage [38]. Seed bulk density and thousand grain weight was determined using the methods of Asghari et al. [39] and Toledo et al. [38], respectively. Ash was measured after igniting the samples in a muffle furnace at 550 °C for 3 h [40]. Protein was determined by a Kjeldahl apparatus [41]. Fiber was estimated according to the way of Muhammad et al. [41]. The ether extract was obtained by a Soxhlet extractor [42]. Total starch was detected by using the Total Starch (AA/AMG) Assay Kit (K-TSTA 04/2009, Megazyme International Ireland Ltd., Wicklow, Ireland) as described by Ahmed and Al-Attar [43]. β-glucan was analyzed using glucose oxidase–peroxidase [44]. The content of Ca in the oat seeds was analyzed by graphite furnace atomic absorption spectrometry [45]. The content of phytic acid and total phosphorus was assayed using the method of iron precipitation and the colorimetric method of yellow phosphorus vanadium molybdate, respectively [45]. All measurements were repeated 5 times, and all experiments were repeated 2 times.

### 2.7. Statistical Analysis

All data were analyzed using SPSS 19.0 (SPSS, Inc., Chicago, IL, USA). The effects of oat variety and LBD (*P. agglomerans*) on panicle length, grains per spike, node number, reproductive branch number, moisture content, bulk density, thousand grain weight, ash, protein, fiber, ether extract, total starch, β-glucan, phytic acid, total phosphorus and Ca were analyzed by two-way ANOVA. A repeated-measures ANOVA with Fisher’s least significant difference (LSD) test was used to determine whether differences between the means were statistically significant. Statistical significance was defined at the 95% confidence level. Means are reported with their standard error. Furthermore, structural equation modeling (SEM) was used to describe the potential causal relationships between explanatory variables, grain yield and nutritional quality and was performed with IBM SPSS Amos 24.0 (Amos Development Co., Ltd. Greene, ME, USA). The strength of direct and indirect relationships between different variables and areas was calculated based on the results of linear regression, and a step-wise fitting procedure was used to achieve the best-supported model based on GFI (goodness of fit index), SRMR (standardized root-mean-square residual) and RMSEA (root-mean-square error of approximation).

## 3. Results

### 3.1. Effects of LBD (P. agglomerans) on Average Disease Infection Rate (ADR) and Average Disease Index (ADI)

**Figure 1:**
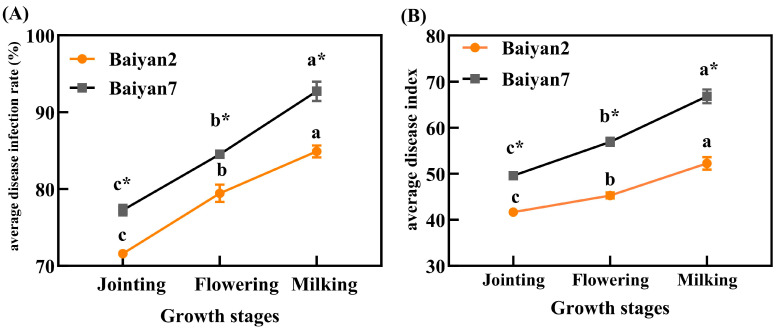
Average disease infection rate (ADR, **A**) and average disease index (ADI, **B**) of LBD in Baiyan 2 (B2) and Baiyan 7 (B7) in jointing, flowering and milking stages. Bars indicate SE, different lowercase letters stand for significant (*p* < 0.05) differences in different growth stages, * indicates a significant difference (*p* < 0.05) between the two varieties.

After inoculating *P. agglomerans* on oat leaves, LBD developed with the growth of the plants. With the development of the oat growing stages, the ADR and ADI for B2 and B7 significantly (*p* < 0.05) increased. Furthermore, ADR and ADI were also influenced by oat variety, and B7 had significant (*p* < 0.05) higher ADR and ADI than B2 in the jointing, flowering and milking stages (Figure 1).

### 3.2. Effects of LBD (P. agglomerans) on Panicle Length, Grains Per Spike, Node Number and Reproductive Branch Number

**Figure 2:**
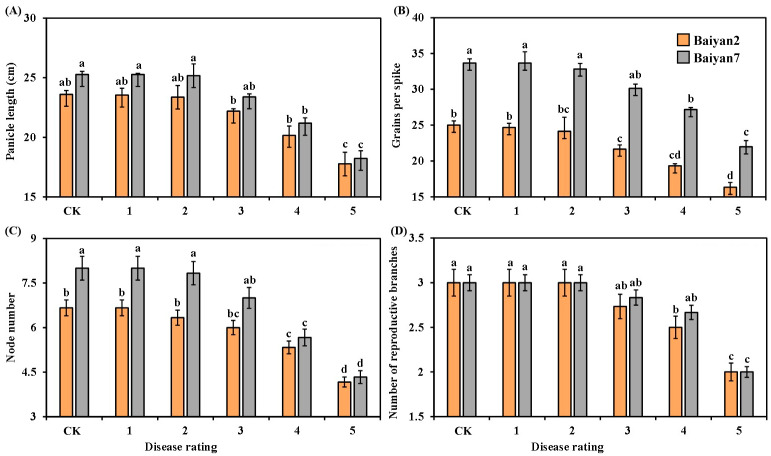
Panicle length (**A**), grains per spike (**B**), node number (**C**) and number of reproductive branches (**D**) for Baiyan 2 (B2) and Baiyan 7 (B7) under 6 disease ratings. Bars indicate SE, and lowercase letters stand for significant differences at *p* < 0.05 (LSD).

Panicle length, grains per spike, node number and reproductive branch number of the two oat varieties were negatively affected by LBD. As the disease rating increased, these parameters for B2 and B7 significantly (*p* < 0.05) decreased, especially for the 4 and 5 disease ratings (Figure 2). There was also a significant (*p* < 0.05) difference between B2 and B7 in grains per spike under different disease ratings, and B7 had a higher number of grains per spike than B2 (Figure 2B). Overall, LBD reduced the panicle length, grains per spike, node number and reproductive branch number of both B2 and B7 by 9.83%, 14.24%, 16.21% and 10.89%, respectively. Otherwise, no significant difference between B2 and B7 was found in panicle length and reproductive branch number under all disease ratings (Figure 2A,D). At the disease rating of 2, the node number of B7 was significantly (*p* < 0.05) higher than that of B2, but there was no difference between B2 and B7 in node number as the disease rating increased from 3 to 5 (Figure 2C).

The effect of oat variety (V) and LBD (D) on the panicle length, grains per spike, node number and reproductive branch number of oat was significant (*p* < 0.05). The interaction effects “V × D” on panicle length and reproductive branch number were also significant (*p* < 0.05), but no significant “V × D” effect on grains per spike and node number was observed (Table 2).

### 3.3. Effects of LBD (P. agglomerans) on Moisture Content, Bulk Density, Thousand Grain Weight and Ash

**Figure 3:**
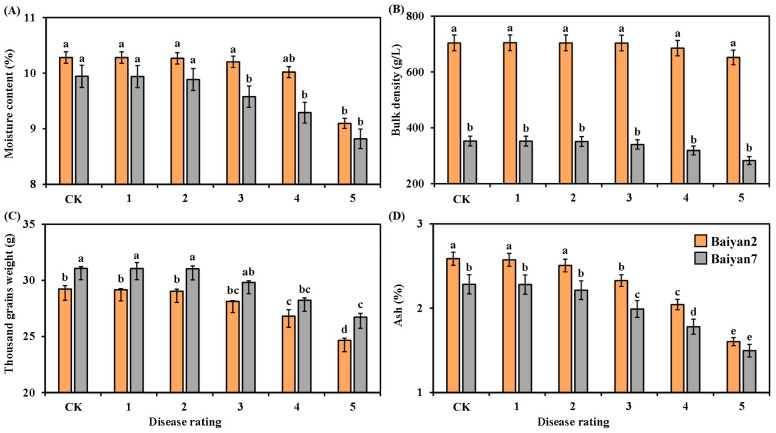
Moisture content (**A**), bulk density (**B**), thousand grain weight (**C**) and ash (**D**) of Baiyan 2 (B2) and Baiyan 7 (B7) under 6 disease ratings. The description of symbols is similar to Figure 2.

Moisture content, thousand grain weight and ash for B2 and B7 changed after LBD development. LBD had significantly (*p* < 0.05) negative effects on moisture content, thousand grain weight and ash of B2 and B7, but there was no significant influence on the bulk density of B2 and B7 (Figure 3). The moisture content and thousand grain weight of B2 and B7 showed no significant difference under CK treatment and in the presence of disease rated 1, 2 and 3, but they were significantly (*p* < 0.05) decreased as the disease rating increased from 4 to 5 (Figure 3A,C). Similarly, no significant difference was observed in ash content between B2 and B7 at the 0, 1 and 2 disease ratings, but as the disease rating increased from 3 to 5, the ash content significantly (*p* < 0.05) decreased (Figure 3D). Compared with uninfected oat, LBD reduced the moisture content, thousand grain weight and ash content of B2 and B7 by 3.71%, 5.57% and 14.52%, respectively. Furthermore, the bulk density of B2 was significantly (*p* < 0.05) larger than that of B7 under all disease ratings, but there was no significant difference between B2 and B7 in moisture content under all disease ratings (Figure 3A,B). Meanwhile, no significant difference between B2 and B7 in thousand grain weight was found under disease rated 0 to 4, but a significant difference appeared at the disease rating of 5 (Figure 3C). The ash content of B2 was higher than that of B7 under all disease ratings (Figure 3D).

Oat variety (V) had a significant (*p* < 0.05) effect on the moisture content, bulk density, thousand grain weight and ash content of the oat seeds. LBD (D) had a significant (*p* < 0.05) effect on moisture content, thousand grain weight and ash content of the oat seeds, but no significant effect was found on bulk density. However, the effect of the interaction of “V × D” on these parameters was evident (*p* < 0.05) (Table 3).

### 3.4. Effects of LBD (P. agglomerans) on Protein, Fiber, Ether Extract and Total Starch in Oat Seeds

**Figure 4:**
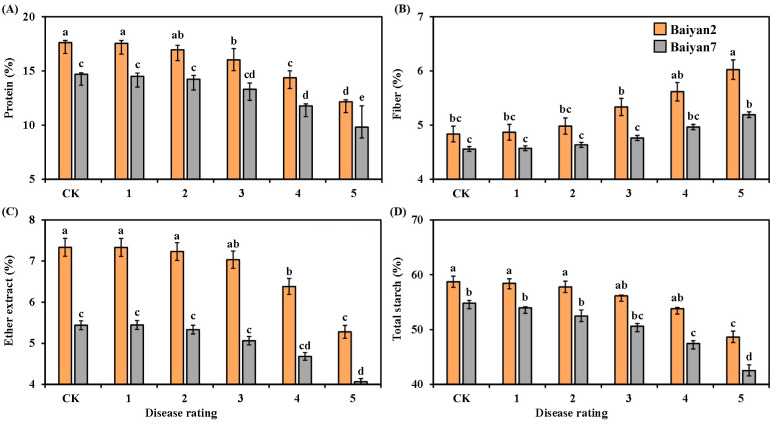
Protein (**A**), fiber (**B**), ether extract (**C**) and total starch (**D**) in Baiyan 2 (B2) and Baiyan 7 (B7) under 6 disease ratings. The description of symbols is similar to Figure 2.

LBD had significantly negative influences on the protein, fiber, ether extract and total starch of B2 and B7. As the disease rating increased from 1 to 5, the contents of protein, ether extract and total starch in B2 and B7 decreased, but the content of fiber increased (Figure 4). Compared with the CK treatment, LBD reduced the average contents of protein, ether extract and total starch in B2 and B7 by 12.9%, 9.45% and 8.12%, respectively. However, the average content of fiber increased by about 8.4%. In addition, the contents of protein, fiber, ether extract and total starch were also affected by the oat variety under different disease ratings. These parameters were higher for B2 than for B7 at all disease ratings (Figure 4).

Both oat variety (V) and LBD (D) had significant (*p* < 0.05) influences on protein, fiber, ether extract and total starch in the oat seeds, and the interaction of “V × D” had also significant effects (*p* < 0.05) (Table 4).

### 3.5. Effects of LBD (P. agglomerans) on β-Glucan, Phytic Acid, Total Phosphorus and Ca in Oat Seeds

**Figure 5:**
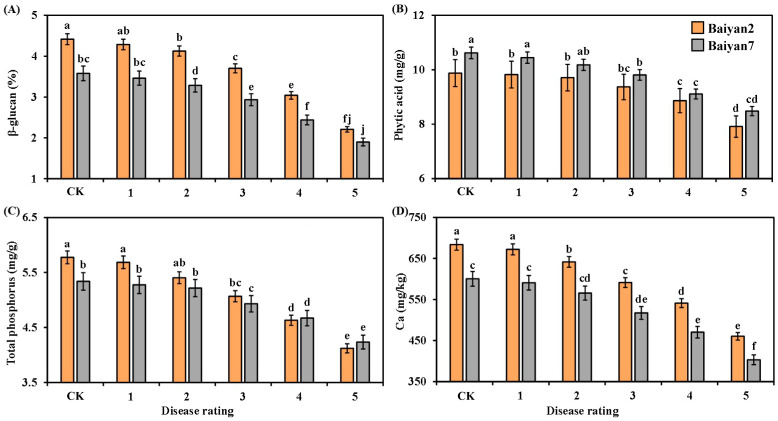
β-glucan (**A**), phytic acid (**B**), total phosphorus (**C**) and Ca (**D**) in Baiyan 2 (B2) and Baiyan 7 (B7) under 6 disease ratings. The description of symbols is similar to Figure 2.

The contents of β-glucan, phytic acid, total phosphorus and Ca in the oat seeds were negatively affected by LBD. With the disease rating increasing from 1 to 5, the β-glucan, phytic acid, total phosphorus and Ca contents in B2 and B7 were reduced (Figure 5). Compared with the CK treatment, the average contents of β-glucan, phytic acid, total phosphorus and Ca in B2 and B7 decreased by 21.51%, 8.52%, 11.29% and 15.06%, respectively. Furthermore, the contents of β-glucan and Ca in B2 were higher than those in B7 at all disease ratings, whereas the phytic acid content of B7 was higher than that of B2 at all disease ratings (Figure 5A,B,D). The total phosphorus content in B2 was significantly (*p* < 0.05) higher than in B7 under CK treatment and grade 1 disease, but no significant difference was found at other disease ratings (Figure 5C).

Oat variety (V) had significant (*p* < 0.05) effects on β-glucan, phytic acid and Ca in the oat seeds, but there was no significant influence on total phosphorus. LBD (D) had significant (*p* < 0.05) effects on β-glucan, phytic acid, total phosphorus and Ca of the oat seeds. However, the effect of the interaction of “V × D” on phytic acid, total phosphorus and Ca was not obvious, but that on β-glucan was significant (*p* < 0.05) (Table 5).

### 3.6. Correlations of LBD (P. agglomerans) and Oat Variety with Grain Yield and Nutritional Quality of Oat

**Figure 6:**
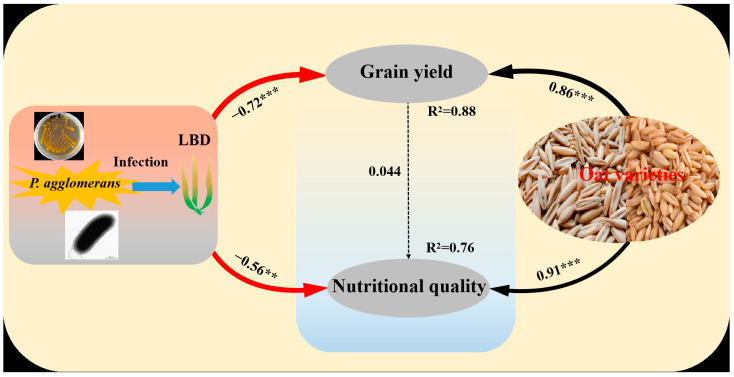
Structural equation model (SEM) showing the effects of variety and leaf blight disease (LBD) on grain yield and nutritional quality of oat. The arrows reflect causal relationships, the numbers close to the lines are standardized path coefficients, the thickness of the black (positive) and red (negative) paths indicates the strength of the relationships. ** *p* < 0.01, *** *p* < 0.001. (CFI = 0.954, RMSEA = 0.074, SRMR = 0.029).

The structural equation model (SEM) respectively explained 88% and 76% of the variation in grain yield and nutritional quality of oat. Meanwhile, the SEM also suggested that LBD (*P. agglomerans*) had significant negative correlations with grain yield (*p* < 0.05) and nutritional quality (*p* < 0.05). However, the variables of grain yield and nutritional quality were significantly positively affected by oat variety (*p* < 0.05). The variable of grain yield had indirect influences on nutritional quality (Figure 6).

## 4. Discussion

The impact of LBD on yield and nutritional quality of oat seeds was first reported in China and other oat-growing areas. Its typical symptoms usually appear as yellow-colored necrotic lesions with water soaking on leaves and eventually causing the leaves to wither and even die. Thus, experiments on the determination of grain yield and nutritional quality after LBD development were conducted to test our hypothesis that LBD has negative influences on the grain yield and nutritional quality of oat. This hypothesis was validated by the obtained results reported in this study. The values of the parameters of panicle length, grains per spike, node number, reproductive branch number, moisture content, thousand grain weight, ash, protein, ether extract, total starch, β-glucan, phytic acid, total phosphorus and Ca in *A. sativa* and *A. nuda* were reduced by LBD. This investigation demonstrated that LBD could decrease the grain yield and nutritional quality of oat seeds. Similar research findings were also showed for rice (*Oryza sativa*), wheat (*Triticum aestivum*) and dry bean (*Phaseolus vulgaris*). LBD separately caused by *Xanthomonas oryzae* [46,47], *Fusarium* spp. [48] and *X. axonopodis* [49] on these crops could result in the loss of grain yield and nutritional quality.

### 4.1. The Grain Yield of Oat Is Decreased by LBD (P. agglomerans)

Disease is a major limiting factor in crop growth and production. Pathogens (e.g., fungi, bacteria and viruses) may cause different pathological changes on host plants, which usually result in grain yield loss and can be transmitted in a field [50,51]. Grain yield is closely related to effective panicle number, panicle length, number of branches per panicle and grains per panicle [52,53,54,55]. In this study, panicle length, grains per spike, node number and reproductive branch number were decreased by LBD. Consistent with our expectations, LBD led to grain yield loss. The extent of yield reduction caused by LBD in the current study was similar to those described by Chowdhury et al. [56] and Lalitha et al. [57]. These studies reported that LBD caused by *Bipolaris sorokiniana* and *Xanthomonas oryzae* in wheat (*Triticum aestivum*) and rice (*Oryza sativa*) significantly reduced plant height, number of tillers, panicle length and grain yield per plant. A possible reason for these decreases is the consumption of carbohydrates by the pathogens [58], which causes a severe decrease in dry matter accumulation in oat seeds and eventually leads to grain yield decrease. Moreover, a previous study showed that this bacterial disease could cause crop leaf yellowing and withering and decrease the leaf area of the seedlings, also decreasing the chlorophyll content and the activity of photosynthesis-related enzymes [36]. As is well known, photosynthesis is the physiological base of plant growth, crop yield formation, and quality improvement, while more than 90% of crop biomass is derived from photosynthetic products [59]. Thus, we consider the reduction in photosynthesis as a consequence of LBD greatly leading to a decrease in oat seed yield and nutritional quality, and suggest that any factor, such as leaf blight, that impairs photosynthesis can lead to reduced grain yield. In our study, this decrease was positively correlated with LBD severity, and the severity of the disease varied among different cultivars. This is similar to previous research that observed that grain yield was negatively correlated with LBD severity, indicating that as the severity of leaf blight increases, grain yield decreases [60]. Another research work showed that LBD led to substantial yield reductions, and the severity of the disease varied among different cultivars, with some experiencing up to a 52.4% reduction in yield [61]. This reduction was primarily due to a decrease in grain number per plant and in thousand grain weight. In conclusion, LBD poses a serious threat to grain formation and yield of oats. Effective management strategies, including the development of resistant cultivars and the use of appropriate fungicides, are essential to mitigate the impact of this disease. These results provide a scientific basis for efficient oat grain production and the control of LBD, hereby contributing to more sustainable production systems of oat.

### 4.2. The Nutritional Quality of Oat Seeds Is Reduced by LBD (P. agglomerans)

The oat grain is most commonly exploited for industrial uses, animal feed, and human nutrition and consumption, which is mainly due to its high yield and nutritional quality (i.e., enrichment in dietary fibers and β-glucan) [62,63]. Meanwhile, its nutritional quality is closely related to seed germination, oat growth, seed health and human food health [64,65]. However, the occurrence and spread of oat diseases can cause nutritional quality loss for the oat grain. Long et al. [66] and Potter [67] reported that crown rust (*Puccinia coronata*) and infection by BYDV can significantly reduce the yield and quality of the oat grain. This is consistent with our findings that revealed that moisture content, thousand grain weight, ash, protein, ether extract, total starch, β-glucan, phytic acid, total phosphorus and Ca of oat seeds were decreased by LBD. A study showed that bacterial diseases also have a significant impact on the nutritional quality of wheat, barley and potatoes, especially the infection by *Fusarium*, which caused a 33–80% decrease in wheat storage protein content and a 14–22% decrease in potato starch content [58,68,69]. This indicates that the degree of harm caused by bacterial diseases is not only closely related to the disease rating, but also influenced by the type of host plant. Moreover, the decrease in the nutritional quality of crops may be due to cellular content losses caused by the pathogen attacking the leaf cells. Therefore, the content of fiber, which is difficult to digest, increases correspondingly [32]. Similarly, our study also showed that the fiber content in seeds increased with pathogen infection. On the other hand, as a consequence of the enzymatic degradation of proteins by pathogens, there is also a considerable decrease in the nutritional value of oat [58]. In addition, some studies showed that the cellular content decreased, and the cellulose levels increased as the disease developed, resulting in reduced nutritional value, which is consistent with our results, as LBD caused nutritional quality loss in oat, and this loss had a positive correlation with LBD severity. This indicates that timely prevention and control measures should be taken in the early stages of disease occurrence to minimize harm. In conclusion, the integration of scientific research into oat production practices can enhance both the nutritional quality and the disease resistance of oat, providing a robust foundation for sustainable agricultural practices.

### 4.3. The Grain Yield and Nutritional Quality of A. sativa and A. nuda Are Different

Both *A. sativa* and *A. nuda* are good spring cereal crops with a high content of protein, fiber, fat and minerals, but their grain yield and nutritional quality are different [70,71,72]. Ma et al. [73] found that the effective tiller number, seed number per panicle and thousand grain weight of *A. sativa* were higher than those of *A. nuda* under different nitrogen fertilizer treatments. However, the nutritional quality of *A. nuda* was higher than that of *A. sativa* under herbicide treatment [72]. Our results showed that *A. sativa* had greater panicle length and higher number of grains per spike, node number, and reproductive branch number than *A. nuda.* In contrast, *A. nuda* had higher contents of protein, total starch, β-glucan, total phosphorus, ether extract and Ca than *A. sativa* with and without LBD. These results align well with those of other studies showing that *A. sativa* had higher leaf area index, total dry matter, and harvest index than *A. nuda* under nitrogen and no-nitrogen conditions [73,74], while the contents of grain crude protein, total phosphorus, Ca, Mg, Zn, Fe and K of *A. nuda* were higher than those in *A. sativa* [75,76]. It was demonstrated that *A. sativa* had higher grain yield than *A. nuda*, but *A. nuda* had higher nutritional quality than *A. sativa* under healthy and diseased conditions, and *A. nuda* had better disease resistance than *A. sativa*. A study showed that oat diseases can be controlled by the use of resistant varieties, specific cultural practices and the application of chemical or biological antimicrobial agents [77]. However, these agents could not be used for a long time and on a large scale because of the resistance, resurgence and persistence problems that they caused. There is increasing evidence that reliance on synthetic chemical pesticides and antimicrobial agents in agriculture has significant negative environmental and health impacts and poses a risk for future food security [78]. Thus, the control of oat diseases through the use of resistant varieties is a well-established and effective strategy and is essential for ensuring food safety and crop productivity. In the current research, we preliminarily explored disease resistance in two oat varieties, and the next step should be to extensively evaluate disease resistance in various oat varieties. In conclusion, the use of resistant oat varieties is a future strategy for the management of oat diseases. Ongoing research and breeding efforts continue to develop new germplasm resources for disease resistance, ensuring the sustainability and productivity of oat crops in the face of evolving pathogen threats, and research is further emphasizing the need for careful cultivar selection based on agronomic practices. The integration of grain yield, nutritional quality, and disease resistance assessments represents a comprehensive approach to selecting oat cultivars, paving the way for a high-efficiency production of oat.

## 5. Conclusions

To our knowledge, this is the first study to investigate the impacts of LBD on grain yield and nutritional quality of *A. sativa* and *A. nuda.* In addition, it is important for understanding the disease damage and losses of grain yield and nutritional quality in oat caused by LBD. This study revealed that LBD had negative influences on the grain yield and nutritional quality of *A. sativa* and *A. nuda.* This was mainly reflected in a reduction in panicle length, grains per spike, node number, reproductive branch number, moisture content, thousand grain weight, ash, protein, ether extract, total starch, β-glucan, phytic acid, total phosphorus and Ca and an increase in fiber in oat seeds. Furthermore, *A. sativa* showed higher grain yield than *A. nuda*, but *A. nuda* had higher nutritional quality than *A. sativa* with and without LBD.

These results suggest that growers who aim to harvest high seed yields should plant B7, and growers who aim to obtain high nutritional quality and disease resistance should plant B2, and timely prevention and control measures should be taken in the early stages of LBD occurrence to minimize its harm and spread and promote the sustainable development of the oat industry.

## Figures and Tables

**Table 1:** Disease rating criteria of bacterial LBD in rice.

Disease Rating	Rating Criteria
grade 0	Asymptomatic
grade 1	Lesion length 2–3 cm
grade 2	Lesion length less than 1/4 of leaf length
grade 3	Lesion length more than 1/4 of leaf length, but less than 1/2
grade 4	Lesion length more than 1/2 of leaf length, but less than 3/4
grade 5	Lesion length less than 3/4 of leaf length

**Table 2:** Results of two-way ANOVA for the effects of variety (V) and disease (D) on panicle length, grains per spike, node number and number of reproductive branches of oat.

Source	df	Panicle Length		Grains per Spike		Node Number		Number of Reproductive Branches	
		F-Value	*p*	F-Value	*p*	F-Value	*p*	F-Value	*p*
V	1	196.983	<0.001	172.456	<0.001	26.691	<0.001	0.131	0.002
D	5	56.751	<0.001	37.327	<0.001	30.422	<0.001	17.767	<0.001
V×D	5	7.786	<0.001	1.052	0.436	1.082	0.415	9.575	<0.001

**Table 3:** Results of two-way ANOVA for the effects of variety (V) and disease (D) on moisture content, bulk density, thousand grain weight and ash of oat.

Source	df	Moisture Content		Bulk Density		Thousand Grain Weight		Ash	
		F-Value	*p*	F-Value	*p*	F-Value	*p*	F-Value	*p*
V	1	38.867	<0.001	191.286	<0.001	87.710	<0.001	5.725	0.022
D	5	4.099	0.006	0.017	1.000	2.295	0.007	31.320	<0.001
V×D	5	7.447	<0.001	21.297	<0.001	2.653	0.022	2.459	0.032

**Table 4:** Results of two-way ANOVA for the effects of variety (V) and disease (D) on protein, fiber, ether extract and total starch of oat.

Source	df	Protein		Fiber		Ether Extract		Total Starch	
		F-Value	*p*	F-Value	*p*	F-Value	*p*	F-Value	*p*
V	1	24.479	<0.001	17.331	<0.001	67.439	<0.001	35.134	<0.001
D	5	198.774	<0.001	57.843	<0.001	2.769	0.036	206.821	<0.001
V × D	5	9.097	<0.001	6.760	<0.001	24.699	<0.001	30.135	<0.001

**Table 5:** Results of two-way ANOVA for the effects of variety (V) and disease (D) on β-glucan, phytic acid, total phosphorus and Ca in oat.

Source	df	β-Glucan		Phytic Acid		Total Phosphorus		Ca	
		F-Value	*p*	F-Value	*p*	F-Value	*p*	F-Value	*p*
V	1	365.654	<0.001	6.169	0.018	2.687	0.110	288.485	<0.001
D	5	21.780	<0.001	30.869	<0.001	67.975	<0.001	18.755	<0.001
V×D	5	1.748	<0.001	1.748	0.122	1.081	0.415	0.623	0.792

## Data Availability

Data will be made available on request.
